# Abdominal paracentesis drainage attenuates severe acute pancreatitis by enhancing cell apoptosis via PI3K/AKT signaling pathway

**DOI:** 10.1007/s10495-020-01597-2

**Published:** 2020-02-25

**Authors:** Chen Luo, Qilin Huang, Xiaohui Yuan, Yi Yang, Bing Wang, Zhu Huang, Lijun Tang, Hongyu Sun

**Affiliations:** 1 Department of Hepatobiliary Surgery, the Affiliated Hospital of Southwestern Medical University, Luzhou, 646000 Sichuan China; 2 Department of General Surgery &, Pancreatic Injury and Repair Key Laboratory of Sichuan Province, The General Hospital of Western Theater Command (Chengdu Military General Hospital), Chengdu, 610083 China

**Keywords:** APD, Severe acute pancreatitis, Apoptosis, PI3K/AKT signaling pathway

## Abstract

Our previous studies have shown that abdominal paracentesis drainage (APD) is a safe and effective strategy for patients with severe acute pancreatitis (SAP). However, the underlying mechanisms behind APD treatment remain poorly understood. Given that apoptosis is a critical pathological response of SAP, we here aim to investigate the effect of APD on cell apoptosis in pancreatic tissues during SAP and to explore its potential molecular mechanism. SAP was induced by 5% sodium-taurocholate retrograde while APD group was inserted a drainage tube into the right lower abdomen of rats immediately after SAP induction. Histopathological staining, serum amylase, endotoxin and inflammatory mediators were measured. Cell apoptosis, apoptosis-related proteins and signaling pathway were also evaluated. Our results demonstrated that APD treatment significantly attenuated pancreatic damage and decreased the serum levels of amylase, endotoxin, TNF-α, IL-1 and IL-6 in rats with SAP. Notably, APD treatment enhanced cell apoptosis and reduced necrosis in pancreatic tissues, as evidenced by Tunnel staining, the increased pro-apoptosis proteins (Cleaved-caspase-3 and bax) and decreased anti-apoptosis protein (Bcl-2). Moreover, the effect of APD on cell apoptosis was further confirmed by the regulatory pathway of PI3K/AKT and NF-kB signaling pathway. These results suggest that APD attenuates the severity of SAP by enhancing cell apoptosis via suppressing PI3K/AKT signaling pathway. Our findings provide new insights for understanding the effectiveness of APD in patients with SAP.

## Introduction

Severe acute pancreatitis (SAP) is a fatal clinical condition usually complicated with systemic inflammatory response syndrome (SIRS) and multiple organ dysfunction syndromes (MODS). So far, the mortality rate of SAP is still up to 20–30% [1]. The key issue is that there is no effective therapy to control the progression of SAP. Pancreatic associated ascites fluid (PAAF) is known to play a critical role in the pathogenesis of SAP because it contains various toxic substances such as tumor necrosis factor, interleukin, endotoxin and so on [2–5]. Our previous clinical and experimental studies showed that abdominal paracentesis drainage (APD) attenuated the outcomes of SAP safely and effectively through removing PAAF [6–10]. However, little is known about the underlying mechanisms responsible for APD treatment for SAP.

Accumulating evidence demonstrates that cell apoptosis represents a favorable response during the course of SAP [11–14]. Apoptosis and necrosis, two forms of cell death, are key pathological responses of SAP. Extensive acinar cell apoptosis was observed in mild acute pancreatitis, whereas acinar cell necrosis with little apoptosis was noted in SAP. That is, apoptosis plays a protective role and is inversely correlated with the severity of SAP [15]. Therefore, the regulation of cell death from necrosis to apoptosis has been suggested to be one of protective mechanisms against SAP [16]. For example, the administration of resveratrol, aspirin and other drugs could significantly reduce the severity of AP by promoting acinar cell apoptosis [17, 18]. Despite these studies, it remains elusive whether APD treatment has influence on cell apoptosis in pancreatic tissues of SAP.

Phosphoinositide 3-kinase (PI3K) has been shown to play a crucial role in the pathogenesis of SAP. It is reported to be implicated in the regulation of multiple pathological responses including calcium signaling alteration, trypsinogen activation, and nuclear factor-κB transcription during SAP [19]. Of note, PI3K signaling pathway modulates acinar cell apoptosis, and regulates inflammatory responses during the course of SAP [20]. Singh et al. reported that PI3K inhibition significantly reduced in pancreatic edema and necrosis in rats with SAP [21]. Other studies have shown that the PI3K/AKT signaling pathway can aggravate the severity of SAP by inhibiting cell apoptosis [22]. However, it is unclear whether APD by draining PAAF would alter the PI3K/AKT signaling pathway and have any association with cell apoptosis.

Based on the above findings, we hypothesized that APD treatment could protect rats from pancreatic injury induced by SAP through enhancing cell apoptosis via PI3K/AKT signaling pathway. To address this hypothesis, we systematically investigated the influence of APD treatment on apoptosis in rats with SAP and determined whether PI3K/AKT signaling play a crucial role during this treatment process.

## Materials and methods

### Reagents and antibodies

Sodium taurocholate was purchased from Sigma Chemical Co. (St. Louis, MO, USA). Antibodies specific for Bax (ab32503) were purchased from Abcam Inc (Cambridge, MA, USA). Antibody for caspase-3 (#9662S), PI3K (4292S), Akt (4691T), p-AKT (Ser473, 4060T), p-AKT (Thr308, 13038T), NF-kBp65 (8242T) was purchased from Cell Signaling Technology (Beverly, MA, USA). Antibody for p-PI3K (PA5-38905) was purchased from Thermo Fisher Scientific Inc (Massachusetts, MA, USA). Antibody for Bcl-2 (GTX100064) was purchased from GeneTex Inc (Irvine, CA, USA). Antibody for GAPDH were purchased from Biosynthesis Biotechnology co. ltd (Beijing, China). Wortmannin was supplied by Solarbio Science Technology co. ltd (Beijing, China). All other chemicals used in this study were of analytical grade and were commercially available.

### Animal and experimental design

The animal protocol was designed to minimize pain or discomfort to the animals. Adult male Sprague–Dawley (SD) rats weighing 200–250 g were purchased from Chengdu Dossy Experimental Animals Co., Ltd. (Chengdu, China). All rats were housed under a 12 h light/dark cycle and pathogen-free conditions. The experimental procedure was approved by the animal protection and use institution committee of the General Hospital of Western Theater Command and in accordance with established international guidelines for animal research.

54 SD rats were divided into 3 groups randomly: SAP group, APD group and sham operation group (SO, n = 18 per group). SAP was induced in the SAP group and APD group by retrograde injecting 5% sodium taurocholate into biliopancreatic duct at the rate of 12 ml/h under standard pressure by using micro-infusion pump (0.01 ml/100 g rat weight), and then the biliopancreatic duct was closed and maintained for 5 min by arterial clip. After puncture needle and arterial clip were removed, the model was successfully constructed following the closure of abdomen. In APD group, a drainage tube with vacuum bulb was implanted in its right lower abdomen immediately after SAP induction [10]. No operation was performed in the SO group except abdominal opening and closing. Isoflurane was used for anesthesia. Rats were put to death at 6 h, 12 h and 24 h respectively after SAP was successfully induced. Blood samples, pancreatic tissues and PAAF were collected and then stored at -80℃ immediately until used.

### Histological analysis of pancreas

Tissue samples were fixed with 4% paraformaldehyde solution. After embedding in paraffin, the tissue samples were cut into about 4 µm thickness, and the sections were stained with hematoxylin–eosin (HE). Light microscope was then used to observe the slide. For assessing the degree of pancreatic injury, the scoring system was utilized according to the reported literature to evaluate several parameters, such as edema, necrosis, bleeding, inflammatory cell infiltration [23]. Five different fields were randomly observed under the microscope each slide.

### Biochemical analysis

Serum TNF-α, IL-1β, IL-6 and endotoxin were detected by Enzyme-linked immunosorbent assay (ELISA) kit (Nanjing Jiancheng Institute of Biological Engineering, China) according to the manufacturer's manual. Serum amylase levels were determined in accordance to the product instructions of the automatic biochemical analyzer (Taikang Technology co. ltd, Jiangxi, China).

### Immunohistochemical staining

Pancreatic tissue of each group was used for immunohistochemistry to detect the expression of Bax, bcl-2 and cleaved-caspase-3 after 12 h of SAP induction. Pancreatic tissues were paraffin-embedded, and then the samples were dewaxed according to the standard procedure. After peroxide-blocking enzyme reaction for 10 min, the sections were placed in 0.01 mol/l sodium citrate solution at constant 100 ℃ for 5 min, and then cooled for 3 min. After that, they were boiled and soaked again for 5 min for antibody repair. 5% bovine serum (Boster Biological Technology co. ltd, Wuhan, China) was used to block for 30 min. The sections were incubated with rabbit derived primary antibody of Bax (1:200), bcl-2 (1:100), caspase-3 (1:200) overnight at 4 ℃. After the primary antibody was washed, sections were incubated with biotin-labeled goat-anti-rabbit immunoglobulin G (Boster Biological Technology co. ltd, Wuhan, China) at 37 ℃ for 30 min. Then they were incubated at 37 ℃ with horseradish peroxidase-labeled streptavidin for 30 min (Boster Biological Technology co. ltd, Wuhan, China). Finally, the target protein was chromogenic reacted with 3,3′-Diaminobenzidine tetrahydrochloride (DAB, Boster Biological Technology co. ltd, Wuhan, China), and the hematoxylin staining was carried out, and then hydrochloric acid alcohol differentiation was followed by ammonia the blue. Pictures are taken under an optical microscope. The brown-yellow region represents cells containing Bax, bcl-2, and cleaved-caspase-3, which were then blinded assessed by two pathologists respectively.

### TUNEL staining

The number of apoptosis cells of pancreatic tissue was detected by TUNEL Apoptosis Detection Kit III, FITC (MK1023, Boster Biological Technology co. ltd, Wuhan, China). According to the manufacturer's instructions, all of the cells showed blue nuclear DAPI staining, but the TUNEL-positive cells displayed green nuclear staining. The stained slices were analyzed by laser-scanning confocal microscopy (Eclipse Ti2; Nikon Instruments, Tokyo, Japan).

### Quantification of necrosis

Necrosis in rat models of pancreatitis was quantified on pancreatic tissue sections stained with H&E. Cells with swollen cytoplasm, loss of plasma membrane integrity and leakage of organelles into the interstitium were considered to be necrotic, as previously described [15, 24].

### Western blot

First, the protein extract kit (Nanjing Jiancheng Bioengineering Institute, China) was used to extract the protein from the pancreas of the rat. Protein concentration was determined by enhanced BCA protein detection kit (Nanjing Jiancheng Bioengineering Institute, China). Equivalent separation (MiniPROTEAN II; Bio-Rad, Hercules, CA, USA) was performed on each protein sample in gel electrophoresis using SDS-PAGE. They were then transferred to a PVDF membrane with bore diameter of 0.22 or 0.40 um. Then blocked with 5% defatted milk or bovine serum (diluted with TBS + tween-20) for 1 h. Primary antibody of bax (1:2000), bcl-2 (1:500), caspase-3 (1:2000), NF-kB (1:1000), AKT (1:1000), p-AKT474 (1:2000), p-AKT308 (1:1000), PI3K(1:1000), and p-PI3K (1:1000) were incubated overnight at 4 ℃. After HRP coupled secondary antibody incubation, chemiluminescence detection reagent (Millipore, Billerica, MA, USA) was added to the membrane, and the membrane was detected with BioSpectrum4 luminescence detector (UVP; Upland, CA, USA). The average optical density (OD) of the corresponding protein bands was recorded, and the ratio of the absorbance value of each protein was calculated.

### Evaluating the effect of PAAF on PI3K/AKT signaling pathway and apoptosis in pancreatic tissues

To measure the effect of PAAF on the activation of the PI3K/AKT signaling pathway and apoptosis in pancreatic tissues, 8-week old male rats were fasted for 12 h then injected intraperitoneally (IP) seven times with saline or cerulein (50 mg/kg) rat weight hourly. 24 rats were randomly divided into four groups (n = 6): (1) control group: rats were intraperitoneally injected with saline. (2) MAP group: MAP rats were intraperitoneally injected with 5 ml normal saline. (3) PAAF reinfusion group (PI): the previously collected PAAF of 24 h SAP group rats were centrifuged, and 5 ml sterile supernatant was intraperitoneally injected into the abdominal cavity of MAP rats. (4) Wortmannin + PAAF group (WP): Wortmannin, a PI3K inhibitor (dissolved in 10% DMSO), was administered by intraperitoneal injection in doses of 1.4 mg/kg, 40 min before 5 ml sterile supernatant was intraperitoneally injected into the abdominal cavity of MAP rats. All rats were anesthetized and killed after 12 h of PAFF infusion. Blood and pancreatic tissues were collected and immediately frozen at – 80 ℃ for storage until used.

### Statistical analysis

Statistics as well as graphical representations were performed using GraphPad Prism™ 8.0 (GraphPad Software Inc., USA). Parametric data are expressed as the means ± SD. Unpaired Student *t* test was used to evaluate the significance between 2 groups. For comparison of more than three groups, one-way analysis of variance (ANOVA) was applied, and nonparametrically distributed variables were compared by the Mann–Whitney U test using SPSS20.0 (SPSS Inc., USA). A value of P < 0.05 was regarded as statistically significant.

## Results

### APD treatment attenuates pancreatic injury in rats with SAP

**Fig. 1:**
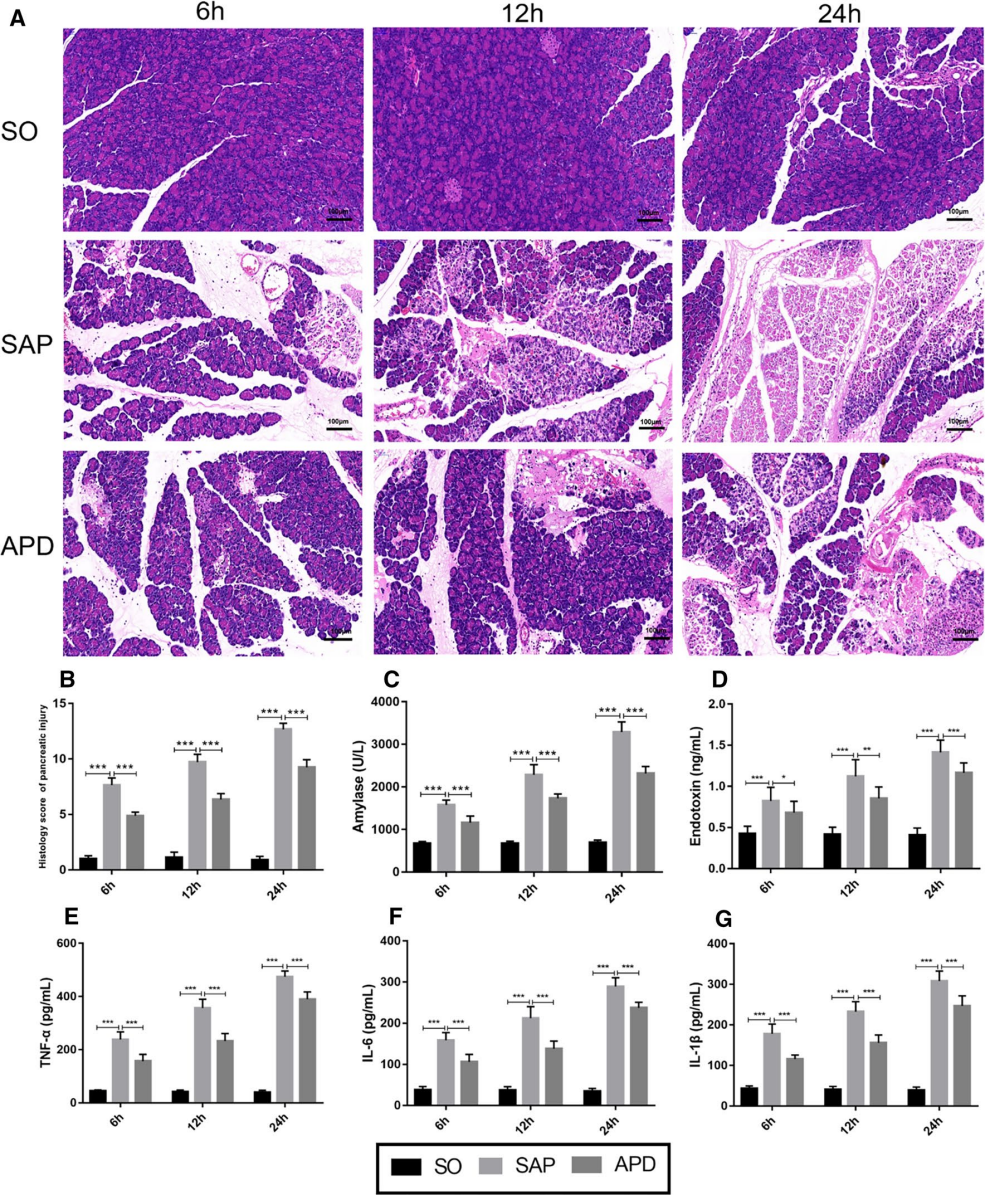
APD treatment effectively alleviate pancreatic injury and systemic inflammatory response. **a** Representative images of rat pancreatic tissue from different groups by HE staining (Bar = 100 µm). **b** Histology score of Pancreatic injury. **c**, **d** Serum amylase and endotoxin. **e**, **f** Serum inflammatory factor (TNF-α, IL-6, IL-1β). All data are presented as mean ± SD (n = 6). SO, sham operation; SAP, severe acute pancreatitis; APD, SAP + APD. *p < 0.05, **p < 0.01, ***p < 0.001

To assess the therapeutic effects of APD on pancreatic injury in rats with SAP, we first performed histopathological scores, and measured serum amylase and inflammatory mediators. Histologically, SAP group showed obvious morphological damage, such as necrosis and inflammatory infiltration, whereas the pancreatic tissue damage was significantly reduced in APD group (Fig. 1a). This result was supported by lower histological scores in APD group than those in SAP group (Fig. 1b). The serum levels of amylase and endotoxin, the hallmark of acute pancreatitis, were also remarkably decreased in APD group compared with SAP group (Fig. 1c, d). Pro-inflammatory cytokines including TNF-α, IL-1β and IL-6, exhibited significant lower levels in APD group than those in SAP group (Fig. 1e–g). These data demonstrate that APD treatment improves tissue damage and reduces inflammation induced by SAP.

### APD treatment enhances cell apoptosis in pancreatic tissues during SAP

**Fig. 2:**
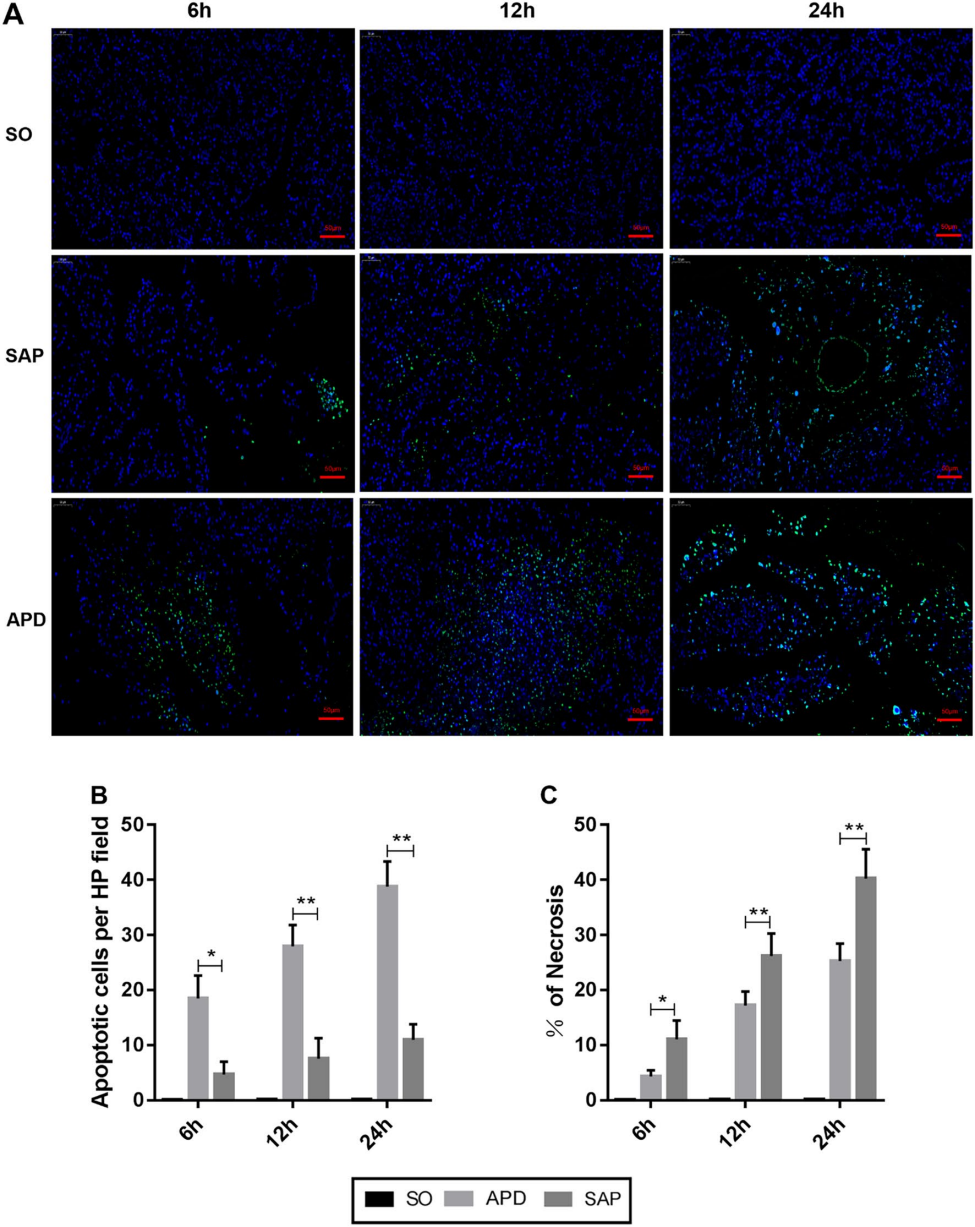
APD treatment increases pancreatic acinar cell apoptosis in rats with SAP. **a** Representative images of pancreatic tissue TUNEL assay (× 400 magnification). **b** Statistical results of apoptotic number of pancreatic acinar cells in each group. **c** Pancreatic necrosis was assessed by HE staining, and pancreatic necrosis was significantly reduced in the APD-treated group compared with the SAP group. All data are presented as mean ± SD (n = 3). SO, sham operation; SAP, severe acute pancreatitis; APD, SAP + APD. *p < 0.05, **p < 0.01, ***p < 0.001

**Fig. 3:**
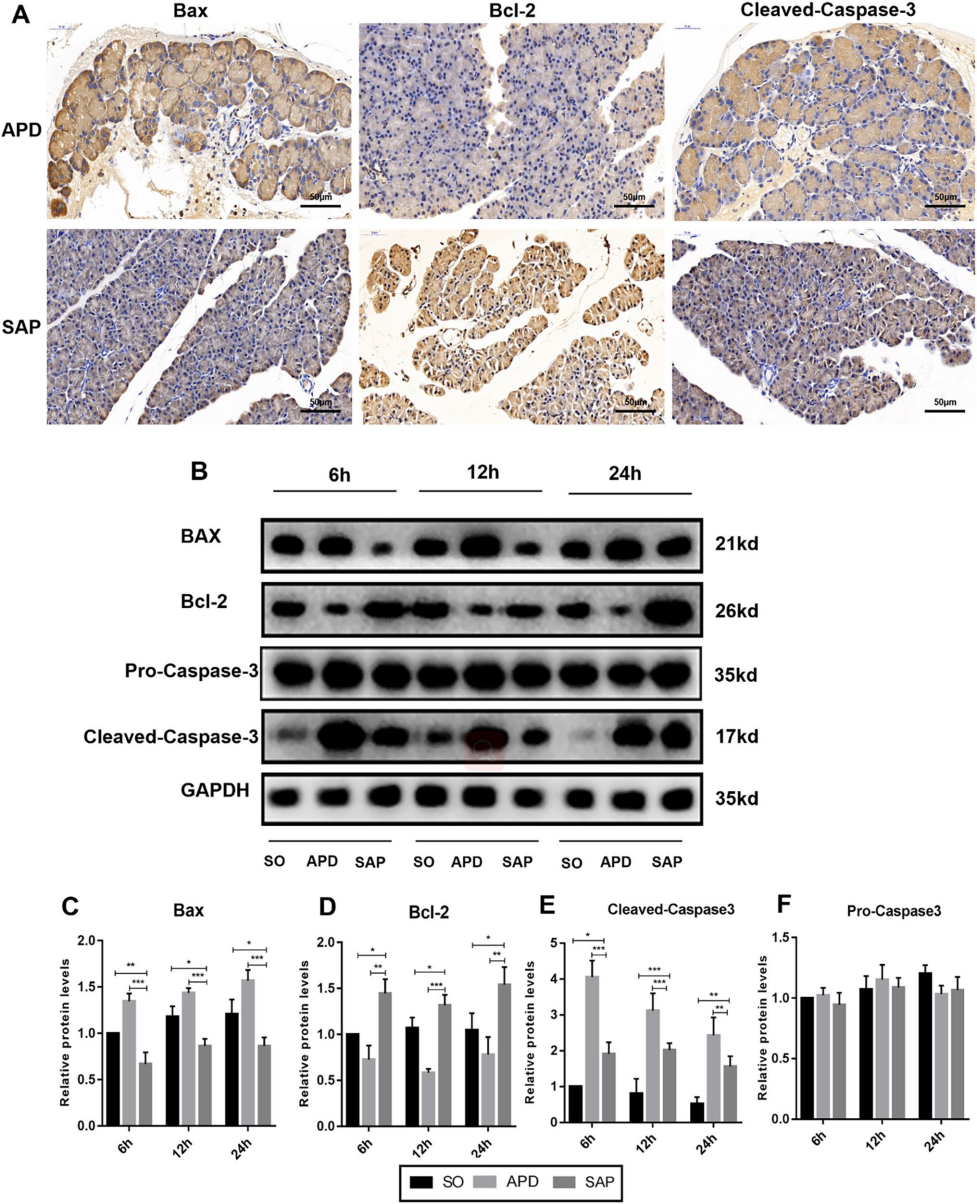
APD treatment enhances pancreatic acinar cell apoptosis in SAP rats. **a** At 12 h after induction of SAP, representative immunohistochemistry images of Bax, Bcl-2 and cleaved-caspase-3 in pancreatic tissue (× 400 magnification). **b** Immunoblotting of pancreatic Bax, Bcl-2, pro-caspase-3 and cleaved-caspase-3 protein expression. **c**–**f** Quantitative densitometric analyses of the immunoblot data of apoptosis-related proteins in pancreatic tissue. All data are presented as mean ± SD (n = 3). SO, sham operation; SAP, severe acute pancreatitis; APD, SAP + APD. *p < 0.05, **p < 0.01, ***p < 0.001

Given that apoptosis is considered as a protective mechanism in the progression of SAP, we wanted to determine whether APD treatment would influence cell apoptosis in pancreatic tissues during SAP. TUNEL staining revealed that the apoptosis index was remarkably higher in SAP group compared with SO group. However, the index of apoptosis in APD treatment group was significantly higher than that in SAP group (Fig. 2a, b). Furthermore, the expression of pro-apoptotic protein Bax, as well as the cleavage of caspase-3, were markedly up-regulated in APD group compared with SAP group by immunoblot experiments (Fig. 3b, c, e). In contrast, the level of anti-apoptotic protein bcl-2 was decreased in APD group than that in SAP group (Fig. 3b, d). This effect of APD treatment on cell apoptosis was further confirmed by immunohistochemical detection, in which Bax, bcl-2 and caspase-3 proteins in pancreatic tissues showed the same trends at 12 h after APD treatment (Fig. 3a). These results suggest that APD treatment increases cell apoptosis in pancreatic tissues during SAP.

### By removing PAAF, APD treatment suppressed PI3K/AKT signaling pathway

**Fig. 4:**
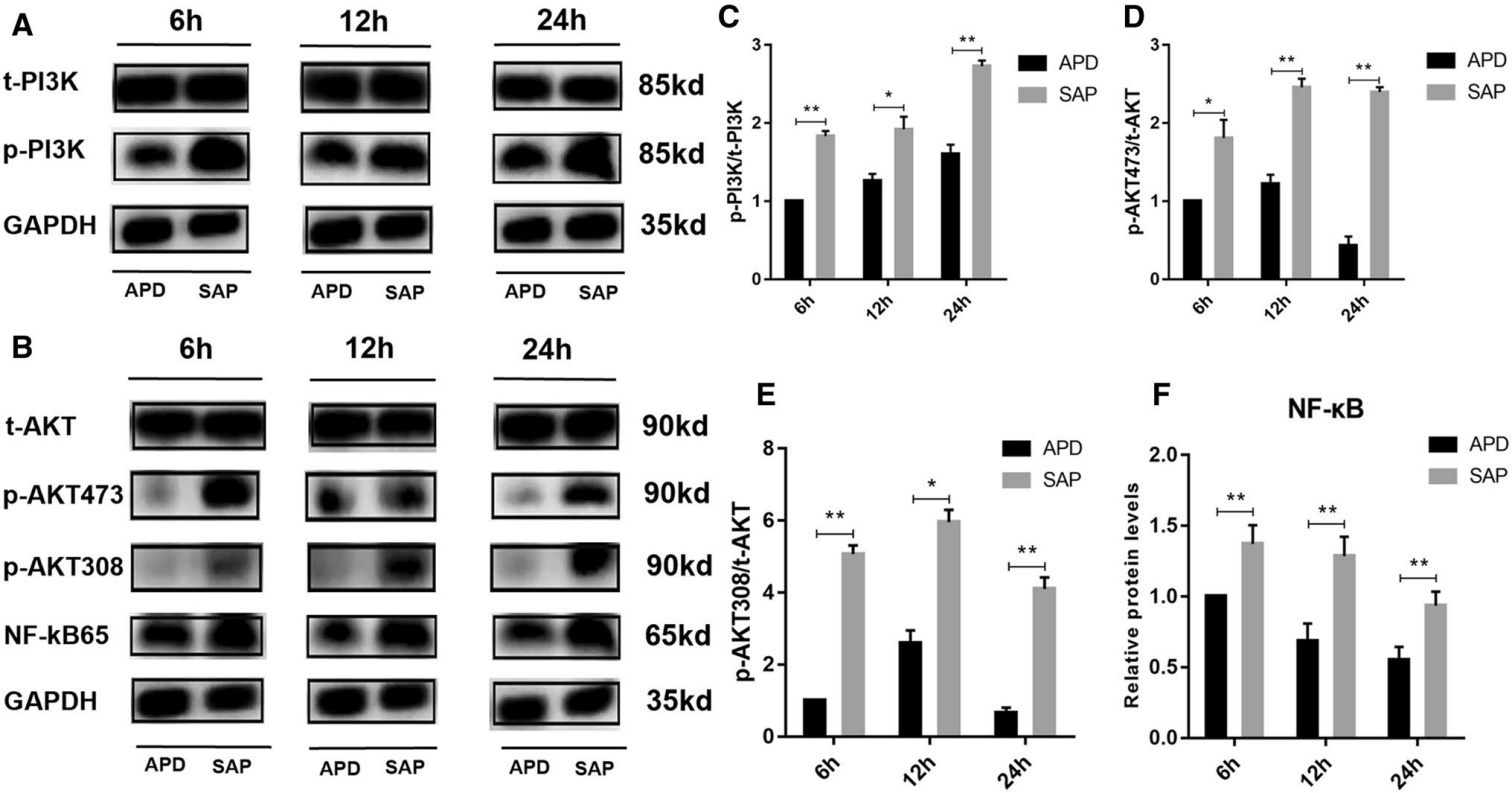
By removing PAAF, APD treatment suppressed PI3K/AKT signaling pathway. **a**, **b** Immunoblotting of pancreatic PI3K, AKT and NF-kb p65 protein expressions. **c**–**f** Immunoblot data were analyzed by densitometry, and the results were presented in histograms respectively. All data are presented as mean ± SD (n = 3). SAP, severe acute pancreatitis; APD, SAP + APD. *p < 0.05, **p < 0.01, ***p < 0.001

Since PI3K/AKT signaling pathway is reported to play important roles in the pathogenesis of SAP, we wanted to explore whether the signaling pathway was involved in the protective effects of APD on pancreatic injury. Through Western blot analysis, we observed markedly decreased expression of p-PI3K and p-AKT in APD group compared with SAP group at various time points from hour 6 to 24 h (Fig. 4a, b), indicating that APD treatment inhibited the activity of PI3K/AKT signaling pathway. Moreover, the expression of NF-kB65, a known downstream signaling molecule, was found to have a significant decrease in APD group than that in SAP at various time points (Fig. 4b, f). These results demonstrate that APD treatment by removing PAAF significantly suppressed PI3K/AKT signaling pathway in SAP rats, suggesting that PI3K/AKT signaling may be responsible for the protective effects of APD on pancreatic injury induced by SAP.

### By removing PAAF, APD treatment attenuates pancreatic injury by suppressing PI3K/AKT signaling pathway

**Fig. 5:**
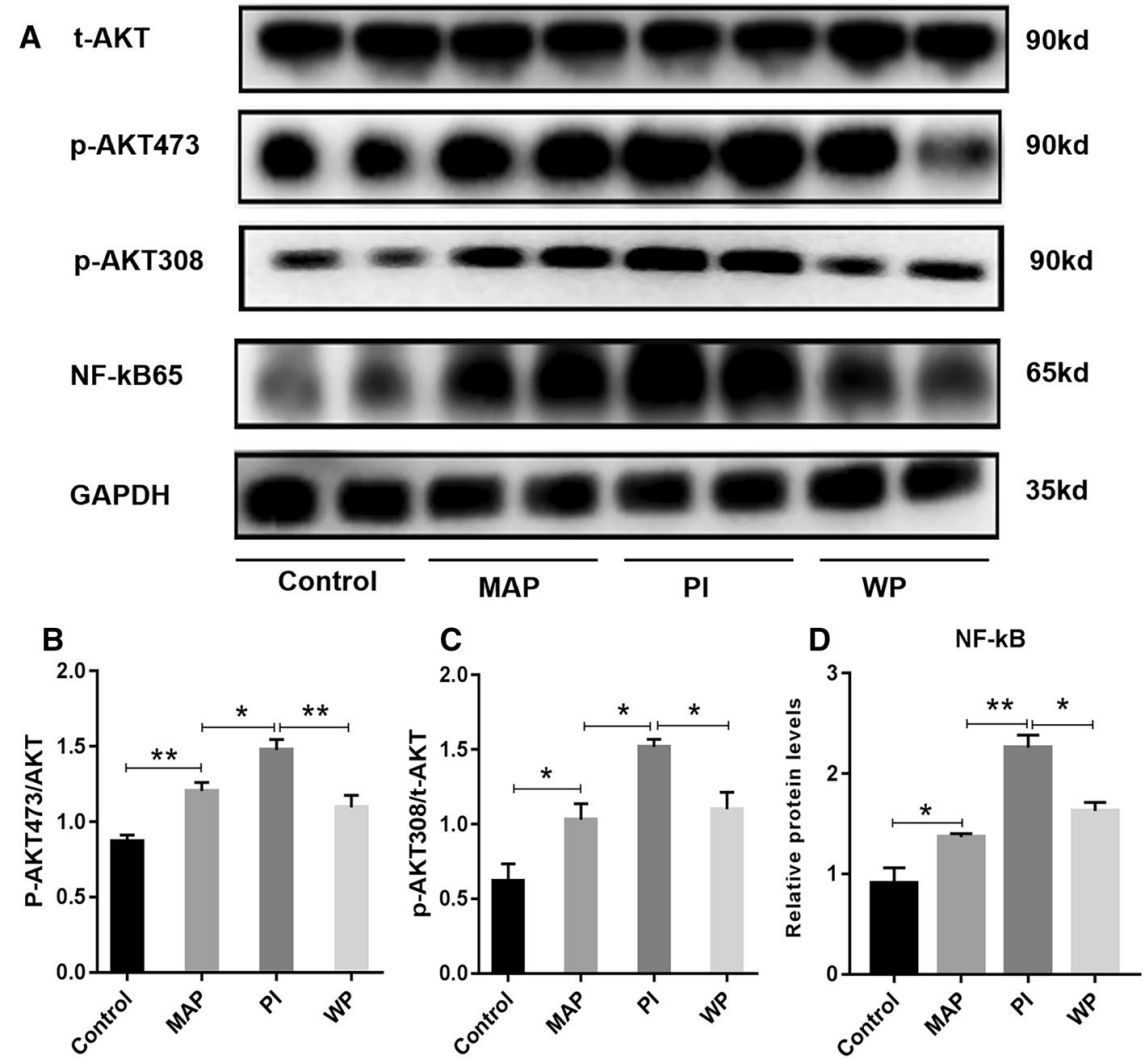
By removing PAAF, APD treatment attenuates pancreatic injury by suppressing PI3K/AKT signaling pathway, **a** Immunoblotting of pancreatic AKT and NF-kb p65 protein expressions. **b**–**d** The blots were analyzed by densitometry, and the results are expressed in the histograms respectively. All data are presented as mean ± SD (n = 3). Con, control; MAP, mild acute pancreatitis; PI, PAAF injection; WP, Wortmannin + PAAF. *p < 0.05, **p < 0.01, ***p < 0.001

Next, we tried to explore whether PI3K/AKT signaling pathway was involved in the protective effects of APD on pancreatic injury induced by SAP. Firstly, we intra-peritoneally injected PAAF or PAAF + PI3K inhibitor (PI and WP groups, respectively) and investigated its influence on PI3K/AKT signaling pathway in rats with MAP. As seen in Fig. 5, the expression levels of p-AKT and NF-κB65 in pancreatic tissues were significantly increased in PI group compared with MAP group. However, a remarkable decrease of p-AKT and NF-κB65 expression was noted in WP group than that in PI group. These results suggest that the administration of PAAF activates PI3K/AKT signaling pathway in pancreatic tissues of acute pancreatitis.

**Fig. 6:**
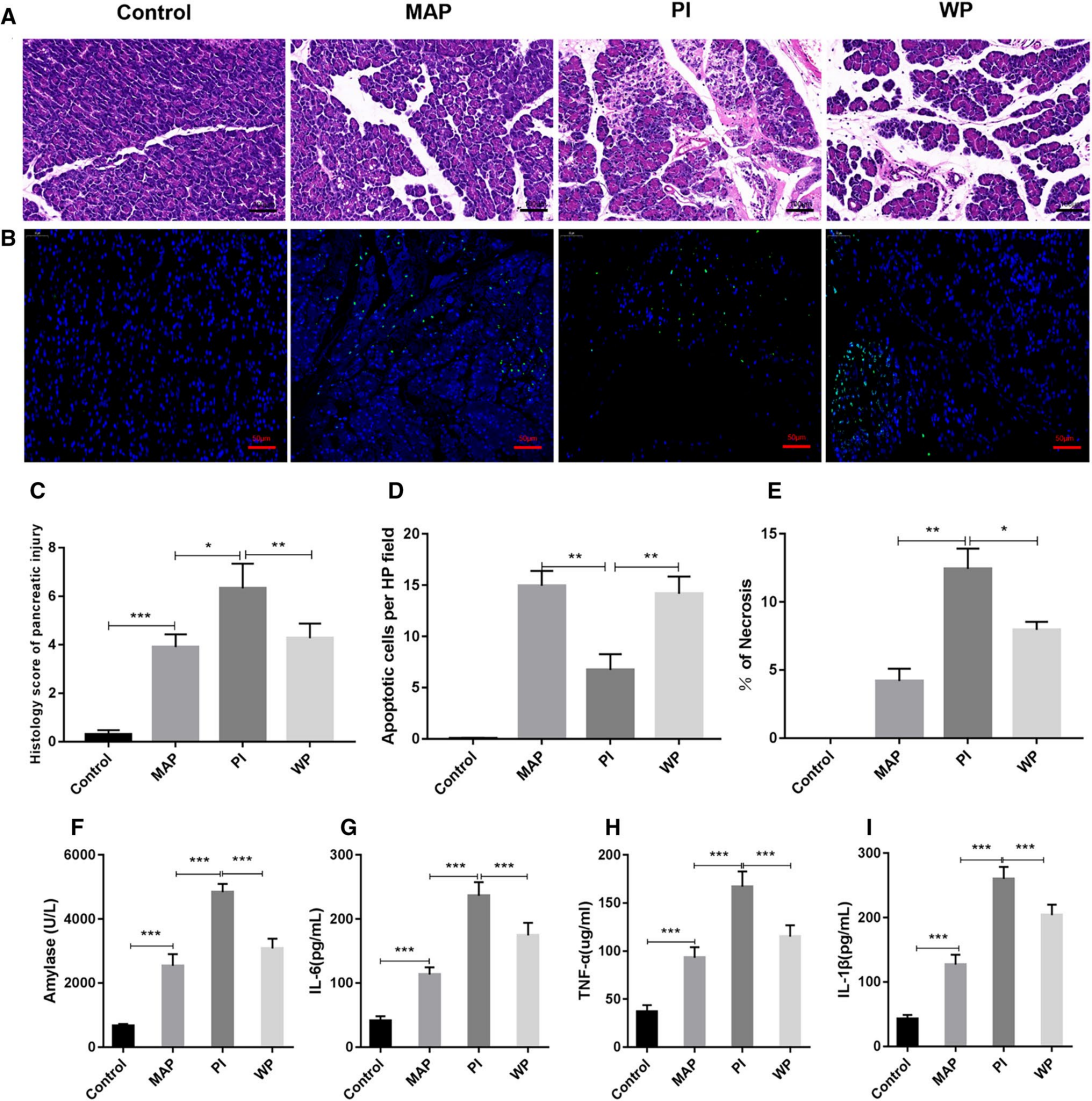
The influence of intra-peritoneal injections PAAF with or without the PI3K inhibitor on two types of pancreatic cell death. **a** Representative micrographs of HE-stained sections of rat pancreatic tissue from different groups (n = 6; × 200 magnification). **b** Representative images of pancreatic tissue TUNEL assay (n = 3; × 400 magnification). **c** Histology score of Pancreatic injury. **d** Statistical results of apoptotic number of pancreatic acinar cells in each group. **e** Pancreatic necrosis was assessed by HE staining. **f**–**i** Serum amylase and inflammatory factor (IL-6, TNF-α, IL-1β) (n = 6). All data are presented as mean ± SD). Con, control; MAP, mild acute pancreatitis; PI, PAAF injection; WP, Wortmannin + PAAF. *p < 0.05, **p < 0.01, ***p < 0.001

Furthermore, we evaluated the influence of intra-peritoneal injections PAAF with or without the PI3K inhibitor on pancreatic injury induced by MAP. HE staining and histopathology scoring showed that the administration of PAAF caused severe pathological changes in pancreatic tissues with obvious necrosis, edema and inflammation (Fig. 6a, c). These severe pathological responses were significantly reduced in pancreatic tissues of rats when treated with the PI3K inhibitor + PAAF. This result was further confirmed by the decreased serum levels of amylase in WP group than those in PI group (Fig.6f). Additionally, pro-inflammatory cytokines like as TNF- a, IL-1β, and IL-6 were also found to be markedly decreased in WP group compared with PI group (Fig. 6g–i). These data suggest that APD treatment by removing PAAF significantly attenuates pancreatic injury by suppressing PI3K/AKT signaling pathway.

### By removing PAAF, APD treatment enhances cell apoptosis in pancreatic tissues by suppressing PI3K/AKT signaling pathway

**Fig. 7:**
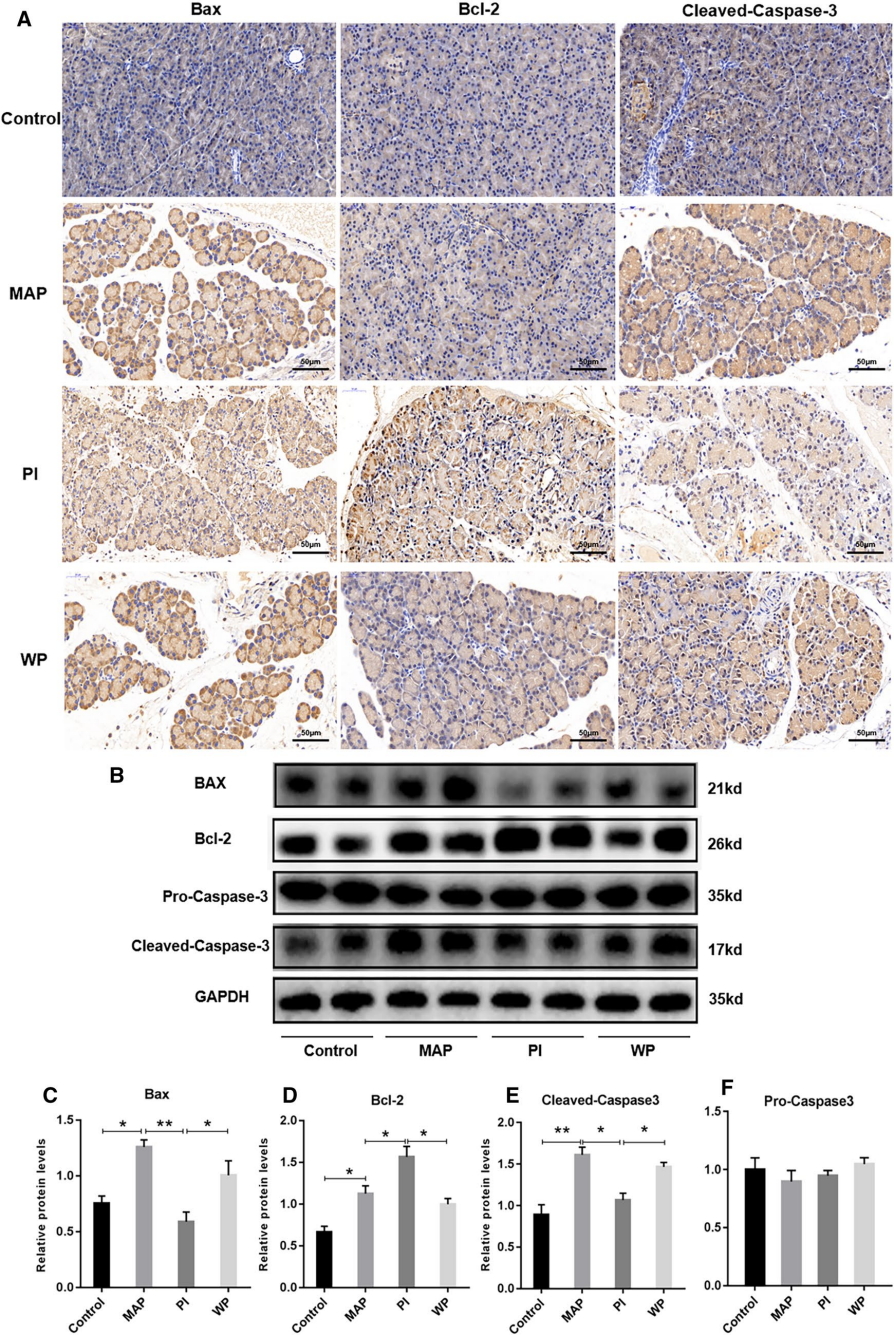
The influence of intra-peritoneal injections PAAF with or without the PI3K inhibitor on PI3K/AKT signaling pathway. **a** At 12 h after modeling for each group, representative immunohistochemistry images of Bax, Bcl-2 and cleaved-caspase-3 in pancreatic tissue (× 400 magnification). **b** Immunoblotting of pancreatic Bax, Bcl-2, pro-caspase-3 and cleaved-caspase-3 protein expression. **c**–**f** Quantitative densitometric analyses of the immunoblot data of apoptosis-related proteins in pancreatic tissue. All data are presented as mean ± SD (n = 3). Con, control; MAP, mild acute pancreatitis; PI, PAAF injection; WP, Wortmannin + PAAF. *p < 0.05, **p < 0.01, ***p < 0.001

Although APD treatment resulted in the increase of cell apoptosis and the suppression of PI3K/AKT signaling in pancreatic tissues during SAP, whether there is any relationship between them remains unknown. To elucidate this point, we firstly evaluated the effects of PAAF on apoptosis of pancreatic tissues in rats with MAP by TUNEL staining. As seen in (Fig. 6b, d), the apoptosis index was strongly lower in PI group than that in MAP group. However, the apoptosis rate in WP group was significantly higher than that in PI group. This result was further confirmed by Western blotting, in which significant changes of Bax, caspase-3 and bcl-2 were noted in WP group than those in PI group (Fig. 7b). Similar results were obtained by immumohistochemical staining (Fig. 7a). These results demonstrate that the administration of PAAF inhibits the apoptosis of pancreatic tissue cells in acute pancreatitis, suggesting that APD treatment by removing PAAF enhances cell apoptosis in pancreatic tissues by suppressing PI3K/AKT signaling pathway.

**Fig. 8:**
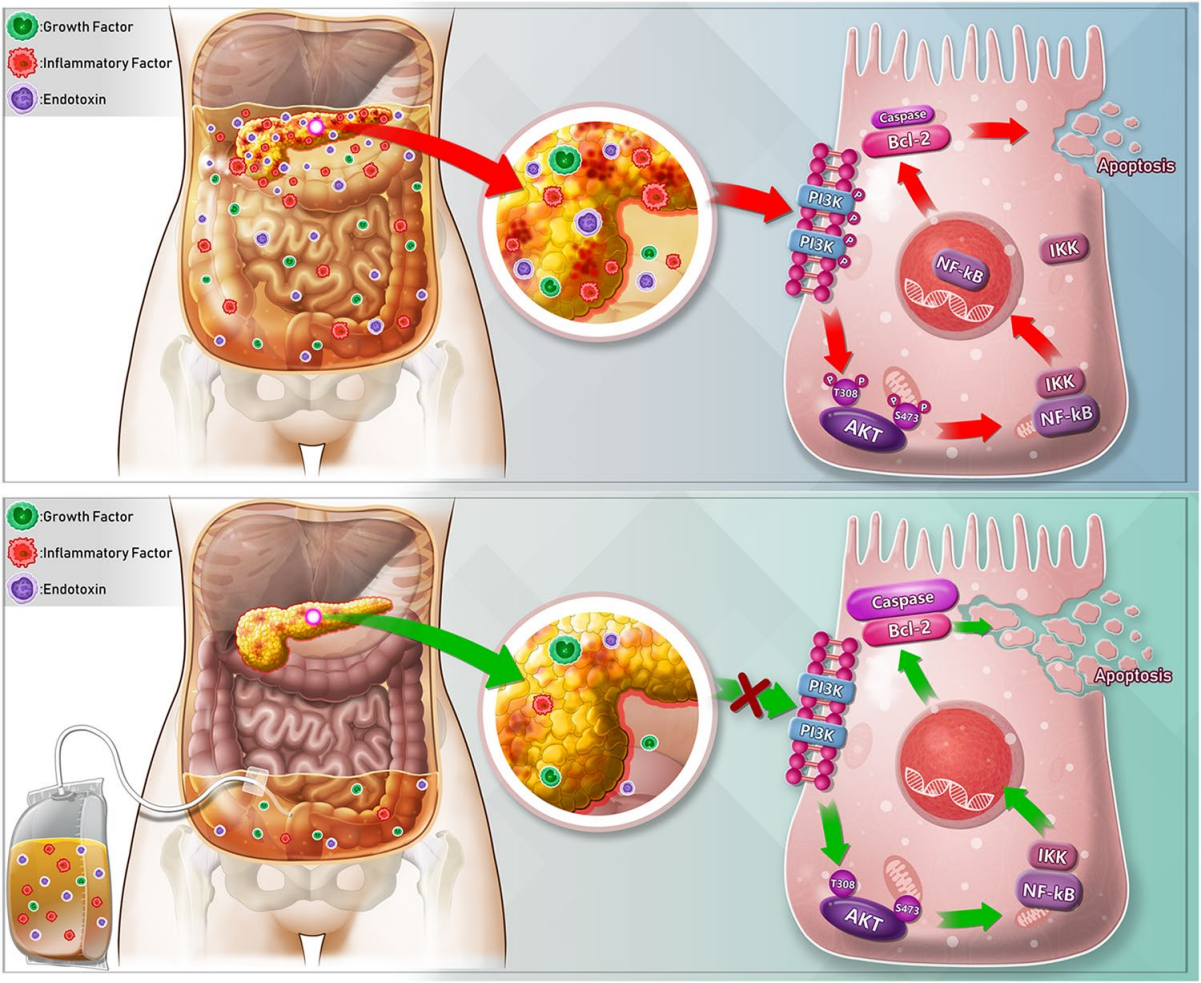
Possible mechanisms responsible for the effect of APD treatment on SAP. Once SAP occurs, toxic substances in PAAF can activate PI3K/AKT signaling pathway in the pancreatic tissue during SAP, which subsequently regulate the expression of apoptosis-related genes via nuclear intervention of the downstream signal molecular NF-κB. These events inhibit cell apoptosis and aggravate the progression of acute pancreatitis. After APD treatment, toxic substances in PAAF were removed, resulting in a significant decline in the activation of PI3K/AKT signaling pathway. The inhibiton of PI3K/AKT signaling will enhance cell apoptosis in pancreatic tissues, and ultimately exerts a protective role on pancreatic injury induced by SAP

Based on the above findings, a schematic model was proposed for the potential mechanism responsible for APD treatment on SAP (Fig. 8). Once SAP occurs, toxic substances in PAAF can activate PI3K/AKT signaling pathway in the pancreatic tissue during SAP, which subsequently regulate the expression of apoptosis-related genes via nuclear intervention of the downstream signal molecular NF-κB. These events inhibit cell apoptosis and aggravate the progression of acute pancreatitis. After APD treatment, toxic substances in PAAF were removed, resulting in a significant decline in the activation of PI3K/AKT signaling pathway. The inhibiton of PI3K/AKT signaling will enhance cell apoptosis in pancreatic tissues, and ultimately exerts a protective role on pancreatic injury induced by SAP.

## Discussion

In the present study, we provide the first evidence that APD attenuates severe acute pancreatitis by enhancing cell apoptosis via PI3K/AKT signaling pathway. The important findings include (i) that APD treatment enhances cell apoptosis in pancreatic tissues by removing PAAF and (ii) that APD treatment suppressed PI3K/AKT signaling pathway and thus enhances cell apoptosis, ultimately attenuating severe acute pancreatitis. These findings provide new insight into the mechanisms responsible for the effectiveness of APD treatment.

As is known, PAAF plays an important role during the progression of SAP. It contains a mass of toxic substances including tumor necrosis factor a, interleukin and endotoxin, which can induce pancreatic tissue necrosis and aggravate the inflammatory response [2–5]. Therefore, various treatment strategies that remove PAAF may be effective in treating SAP [25]. In a series of our previous clinical reports, APD treatment have been confirmed in reducing circulating inflammatory factor levels, delaying or avoiding multiple organ failure, reducing mortality and without increasing infection rates [26]. All these indicate that APD is a safe and effective strategy for treating SAP patients. In this study, we confirmed that APD treatment not only decreased inflammatory factors and the amylase level in serum, but also relieved the extent of pancreatic tissue injury, which was consistent with our previous reports.

A large number of studies reveal that apoptosis or necrosis has a significant influence during the course of SAP. In the early stage of SAP, various factors like as TNF-α and IL-1β can cause acinar cell death that occurs mainly via apoptosis or necrosis [27]. Acinar cell necrosis increases pancreatic enzyme activation in pancreatic tissue, and releases multiple inflammation mediators to trigger SIRS. Interestingly, acinar cell apoptosis, in contrast, reduced the activation and release of pancreatic enzymes, thereby avoiding the occurrence of SIRS [15]. Therefore, it is generally accepted that necrosis is a trigger for inflammation, while apoptosis has a protective effect on acinar cells. In this study, we found that APD treatment markedly enhanced the apoptosis index in pancreatic tissues through TUNEL staining, which is consistent with the literature reported by Lu et al. [18]. This result was supported by immunoblot analysis, in which cleaved-caspase-3 and Bax were significantly increased, and bcl-2 was apparently decreased after APD treatment. Among them, caspase-3 is an effector in the caspase family and a key enzyme regulating the apoptosis process, when caspase-3 cleaved into cleaved-caspase-3, it was activated to regulate the process of apoptosis. Interestingly, Bax protein acts contrary to bcl-2 protein. When Bax forms homologous dimer, the induction of apoptosis increases. When Bax and bcl-2 form heterodimers, the anti-apoptotic function of bcl-2 is activated and apoptosis is inhibited [28–30]. Furthermore, the administration of PAAF were found to have a significant decrease in cell apoptosis in pancreatitis of mild acute pancreatitis models, which indirectly indicates that APD treatment by the removal of PAAF promotes pancreatic cell apoptosis in SAP rats, thereby reducing pancreatic necrosis and attenuating the severity of SAP.

PI3K signaling pathway has been shown to play important roles not only in the pathogenesis of SAP but also in the regulation of apoptosis signaling [31]. It can be activated by various factors, such as TNF-α, IL-1β, endotoxin, etc. [32–34], which are also released during SAP. It is reported that PI3K/AKT signaling pathway can exacerbate diseases by inhibiting pancreatic cell apoptosis during the development and progression of SAP [35]. After applying the relevant enzyme PAG synthesized by hydrogen sulfide on SAP rats, Wang et al. found that after inhibiting the activation of PI3K signaling pathway, the activity of the downstream signal molecule NF-kB significantly reduced, thereby increasing the expression of pro-apoptotic genes, and the SAP condition was significantly reduced [36]. In this study, signal molecules such as PI3K, AKT and NF-kB were detected by Western blotting. The phosphorylation levels of PI3K and AKT and the expression of NF-kB were significantly lower after APD treatment, indicating that APD inhibited PI3K/AKT signaling pathway, thereby reducing the involvement of downstream NF-kB in the nucleus and reducing the inhibition on pancreatic cell apoptosis, and ultimately exerted a role in the treatment of acute pancreatitis. This is consistent with the results reported by Lupia et al. [21]. In order to further verify the relationship between the enhanced apoptosis and the inhibited PI3K/AKT signaling, we administrated PAAF or PI3K inhibitor + PAAF into mild acute pancreatitis. When PI3K inhibitor Wortmannin was given in advance and then PAAF was injected into the abdominal cavity, the phosphorylation level of AKT and expression of NF-kB were notably reduced compared with the PAAF treating only, and the number of pancreatic cell apoptosis was significantly increased, while the aggravation of pancreatitis was not obvious. These data indicate that APD treatment can inhibit the overactivation of PI3K/AKT signaling pathway through draining toxic substances from PAAF, thereby enhancing the increase of pancreatic cell apoptosis, and ultimately lighten the severity of SAP.

Our study has several limitations. Firstly, our results are obtained by the model of sodium-taurocholate induced SAP, and the different models of SAP should be utilized in the future experiments. Secondly, as for which kind of toxic substance activate PI3K/AKT signaling pathway during SAP are still unknown, future study should be carried out to clarify the precise mechanisms underlying the protective effect on pancreatic damage induced by SAP.

In conclusion, our study found that APD treatment by removal of PAAF caused the suppressed PI3K/AKT signaling pathway, resulting in pancreatic cells death from necrosis towards apoptosis, and ultimately facilitated the improved outcome of SAP.

## Abbreviations

AKT(PKB): Protein kinase B
ANOVA: One-way analysis of variance
APD: Abdominal paracentesis drainage
Bcl-2: B-cell lymphoma 2
Caspase3: Cysteine aspartic acid protease 3
ELISA: Enzyme-linked immunosorbent assay
IL: Interleukin
MAP: Mild acute pancreatitis
MODS: Multiple organ dysfunction syndrome
NF-kB: Nuclear factor kappa-light-chain-enhancer of activated B cells
PAAF: Pancreatitis associated ascitic fluids
PI3K: Phosphatidylinositide 3-kinases
SAP: Severe acute pancreatitis
SIRS: Systemic inflammatory response syndrome
TNF-α: TNF-α
